# Comparative Assessment of the Phytochemical Composition and Biological Activity of Extracts of Flowering Plants of *Centaurea cyanus* L., *Centaurea jacea* L. and *Centaurea scabiosa* L.

**DOI:** 10.3390/plants10071279

**Published:** 2021-06-23

**Authors:** Natalia Sharonova, Evgeny Nikitin, Dmitriy Terenzhev, Anna Lyubina, Syumbelya Amerhanova, Kseniya Bushmeleva, Adelya Rakhmaeva, Igor Fitsev, Kirill Sinyashin

**Affiliations:** 1 Federal State Budgetary Institution of Science Federal Research Center «Kazan Scientific Center of Russian Academy of Sciences», ul. Lobachevskogo, 2/31, 420111 Kazan, Russia; berkutru@mail.ru (E.N.); dmitriy.terenzhev@mail.ru (D.T.); aplyubina@gmail.com (A.L.); syumbelya07@mail.ru (S.A.); ks.bushmelewa09@ya.ru (K.B.); ermakowa.adelya@yandex.ru (A.R.); sinkirol@mail.ru (K.S.); 2 Federal State Budgetary Scientific Institution «Federal Center for Toxicological, Radiation, and Biological Safety», Nauchny Gorodok-2, 420075 Kazan, Russia; fitzev@mail.ru

**Keywords:** *Centaurea cyanus* L., *Centaurea jacea* L., *Centaurea scabiosa* L., extract, flowers, phytochemical composition, antimicrobial activity, phytopathogenic microorganisms, antioxidant activity, phytotoxicity

## Abstract

The data on the phytochemical composition and biological activity for flowering plant extracts of the genus *Centaurea* (Knapweed)—cornflower (*Centaurea cyanus* L.), brown knapweed (*Centaurea jacea* L.), and greater knapweed (*Centaurea scabiosa* L.), which are typical representatives of the flora in the middle belt of the Russian Federation, were obtained. For the first time, biologically active substances such as pyranone, coumaran (2,3-dihydrobenzofuran), and 5-hydroxymethylfurfural were identified in ethanol and methanol extracts of *Centaurea scabiosa* L. by gas chromatography–mass spectrometry. Catechol and α-amyrin were the major components of the ethanol extract from *Centaurea cyanus* L., and flavone was the major component of *Centaurea jacea* L. flower extract. The greatest antimicrobial activity against phytopathogens was detected in *Centaurea scabiosa* L. when extracting freshly harvested flower biomass with methyl tert-butyl ether at room temperature: the minimum inhibitory concentrations were 60–120 µg/mL, the minimum fungicidal concentration was 120 µg/mL, and the minimum bactericidal concentration was 250 µg/mL. The low antioxidant activity of the studied plant extracts was established using the maximum values of *Centaurea jacea* L. Ethanol extract of *Centaurea cyanus* L. flowers had low antimicrobial and antioxidant activity. The extracts showed no phytotoxicity to garden cress germination but inhibited the growth of juvenile plants, especially roots. The greatest phytotoxic effect was revealed with methyl tert-butyl ether, where the depression of growth indicators was 35% or more.

## 1. Introduction

The global market for organic plants has undergone rapid growth in recent years. However, the range of available and effective plant protection products approved for organic agricultural production is insufficient. One of the more promising and environmentally safe preparations comprises components based on plant raw materials—biologically active plant extracts with inhibitory and/or biocidal effects against phytopathogens. Wild species of the Asteraceae family, one of the largest and most important plant families, are of particular interest for use as a source of active ingredients [1,2]. Currently, the family has 32,913 recognized species divided into 1911 genera in 13 subfamilies [3]. Plants of this family are widespread throughout the world; many have proven therapeutic potential and contain a wide range of biologically active compounds [4].

*Centaurea* L. is a genus of herbaceous perennial plants (although occasionally biennial or annual plants of the Asteraceae family do occur), including more than 700 species [5]. These species are widely distributed throughout Europe, North Africa, South and North America, and a large part of Asia. About 100 species grow in the territory of the Russian Federation [6]. Knapweeds grow in steppe and forest areas, floodplains and dry meadows, deposits, margins of fields, and meadow slopes. The stocks of some species are quite significant.

The genus *Centaurea* L. is widely used in traditional medicine. The species *Centaurea cyanus* L. is included in the Russian Federation’s current pharmacopeia; its flowers are used as a diuretic. *Centaurea cyanus* L. and *Centaurea scabiosa* L. are also used as a diuretic and tonic in Scottish medicine [5]. *Centaurea pulchella*, *Centaurea drabifolia*, and *Centaurea solstitialis* are used to treat abscesses, hemorrhoids, peptic ulcers, and colds in Turkish traditional medicine [7].

The biological activity of extracts and essential oils of different types of *Centaurea* L. has been established, and includes antitumor, antidiabetic, anti-inflammatory, analgesic, antidepressant, antiplasmoid, antirheumatic, antioxidant, antimicrobial, and enzymatic properties [1,2,4,5,8,9,10].

The chemical composition of *Centaurea* plants varies greatly depending on the species and its range. The most characteristic biologically active components are sesquiterpene lactones [11,12], flavonoids, lignans, alkaloids [9], phenolic compounds [13,14], steroids, and terpenes, etc. [4,15,16].

Notably, *Centaurea* L. is still an understudied species both pharmacologically and chemically. Phytochemical composition data on many species are either missing or incomplete.

The goal hereof was to a collect knowledge base on the state of the art regarding the phytochemical composition and biological activity of extracts produced from *Centaurea cyanus* L., *Centaurea jacea* L., and *Centaurea scabiosa* L., which are typical to the Russian Federation, to assess their potential for use in organic agriculture. To that end, the researchers pursued the following objectives: (1) to investigate the phytochemical composition of extracts for the detection and identification of active compounds; (2) to test the antimicrobial activity of the extracts against phytopathogenic bacteria and fungi strains; (3) to test the antioxidant activity of the extracts; and (4) to evaluate the phytotoxic effect of the extracts on seed germination and the parameters of the initial growth of the test plant.

## 2. Materials and Methods

### 2.1. Plant Material

*Centaurea* L. plants were harvested in the Verkheuslonsky Municipality in the Republic of Tatarstan (Russian Federation) in the summer of 2020. The plants were then identified by Dr. Firdaus Khazieva (All-Russian Research Institute of Medicinal and Aromatic Plants, Moscow, Russia). Samples were submitted to the same institution’s herbarium for storage. The above-ground parts of the plants were harvested at the flowering stage. The flowering plants were harvested at the flowering stage in samples of 500 g for further analysis.

### 2.2. Making Centaurea L. Extracts

Flower extracts were produced from freshly harvested and flash-frozen biomass by a single maceration process. In the former case, freshly harvested flowers were detached from stems and ground using a Russian-made LM 202 mill. A sample weighed out to 15 g of the ground biomass was admixed with: (1) 150 mL of water; (2) 150 mL of 70% ethanol; (3) 150 mL of methanol (chemically pure); (4) 150 mL of methyl tert-butyl ether (MTBE) (chemically pure); and (5) 150 mL of petroleum ether (chemically pure) and was then macerated over 1.5 h at 45 °C with continuous stirring. Alternatively, the flower biomass was flash-frozen through 30-min exposure to liquid nitrogen, and then ground to make powder with a pre-cooled mortar and pestle. The subsequent extraction procedure was the same as for freshly harvested flowers. Then the extracts were macerated over 1.5 h at 45 °C with continuous stirring. A total of 10 different types of extracts were obtained, each in triplicate. The resulting mixtures were filtered using a Whatman 1 unit, and then the filtrate was concentrated using a LabTexRe 100-Pro rotor vaporizer. The extracts were stored in dark at 4 °C.

### 2.3. Gas Chromatography–Mass Spectrometry Analysis

The mass spectra of the extracts were recorded using a Trace 1300 chromatographer equipped with a DSQ mass-selective detector (Thermo Fisher Scientific Inc., Waltham, MA, USA); El, 70 eV, m/z = 30–550; CI, 30 eV, m/z = 100–550. Qualitative and quantitative gas chromatography–mass spectrometry (GC-MS) analysis was performed using a TraceGold TG-5 MS fused silica column (30 m × 0.25 mm × 0.25 μm, Thermo Fisher Scientific Inc., Waltham, MA, USA). Conditions of GC split were as follows—injector temperature: 280 °C, interface temperature: 280 °C, initial thermostat temperature: 70 °C, rate of temperature elevation in the column: 10 °C/min, final temperature of the column thermostat: 280 °C, splitless injection, sample size: 1 μL, and volume velocity of gas vehicle (He, 99.999%): 0.9 mL/min at a constant flow rate.

Mass-spectral data were processed on the Xcalibur software (Thermo Fisher Scientific Inc., Waltham, MA, USA) using the NIST’17 electronic mass-spectrum library, NISA MS Search Program V. 2.3, and NIST MS Interpreter (NIST, USA).

Regression analysis of calibration characteristics and mathematical forecasting of the peak area response were performed using Origin V. 6.1 software (OriginLab Corp, Northampton, MA, USA), and processed according to the standards [17,18]. The following reagents were also used: hexane, acetonitrile, and methanol for HPLC. For solid-phase extraction, the team used Diapak C18 Plus concentration cartridges (BioChemMac, Moscow, Russia) which feature increased capacity and selectivity. These cartridges are based on a sorbent with chemically bonded octadecyl groups.

### 2.4. UHPLC-ESI/HRMS Analysis

UHPLC-ESI/HRMS were acquired on LC/HRMS system consisting of a Q Exactive Plus (Thermo Fisher Scientific Inc., Waltham, MA, USA) mass spectrometer, equipped with a heated HESI-II source coupled to a UHPLC system Dionex Ultimate 3000RSLC (Thermo Fisher Scientific Inc., Waltham, MA, USA). Chromatographic separation was achieved on an AkzoNobel Kromasil ExternityXT-1.8-C18 (Bohus, Sweden) narrow-bore column (2.1 × 100 mm, 1.8 μm), equipped with Phenomenex Security Guard ULTRA UHPLC EVO C18 (Torrance, California, USA) and maintained at 40 °C. The instrument parameters for negative mode were as follows: spray voltage 2.5 kV, sheath gas 38 psi, and auxiliary gas 12 a.u., while all other parameters were the same as in positive mode. Mass resolution in full scan mode in the mass range m/z 100–1500 was set to 70,000 FWHM (at m/z 200), while in data-dependent MS/MS the resolution was 17,500 FWHM (at m/z 200), and a 1.0 amu isolation window of precursor ions was used for structural elucidation studies. All solvents were of LC-MS grade and were purchased from Thermo Fisher Scientific (Waltham, MA, USA). The proposed structures were theoretically studied using Mass Frontier 5.1 Software (Thermo Fisher Scientific Inc., Waltham, MA, USA).

### 2.5. Antimicrobial Tests

#### 2.5.1. Microorganism Strains and Nutrient Media

The following phytopathogenic strains were used: bacteria *Agrobacterium tumefaciens* A-47, *Erwinia amylovora* S59/5, *Erwinia carotovora spp. carotovora* SCC3193, *Pantoea agglomerans, Pseaudomonas syringae pv. atrofacience, Xanthomonas arboricola* S3, *Clavibacter michiganensis* VKM Ac-1404, and fungi *Alternaria solani* K-100054, *Fusarium graminearum, Fusarium culmorum*, and *Phytophtora sp*., and the human pathogen strain *Staphylococcus aureus* 209P. Microorganisms were cultured in standard sterile nutrient broths. Bacteria concentrations were detected through standard protocols using a DEN-1B densitometer (Biosan, Riga, Latvia).

Norfloxacin (Sigma-Aldrich Co., St. Louis, Missouri, USA), chloramphenicol (Kazan Pharmaceutical Plant, Kazan, Russia), and difenoconazole (Score 250 EC, Syngenta, Basel, Switzerland) were used as reference compounds in experiments.

#### 2.5.2. Antimicrobial Tests In Vitro

The experiments were designed to find the minimum inhibitory concentration by 2-fold sequential dilution [19] in the modification [20]. The fungistatic activity of the tincture was tested by serial dilution [21] in a liquid medium.

Liquid broth with microbial spores was prepared using standard nutrient media: Hottinger broth for *Staphylococcus aureus* 209P, Potato Extract Glucose broth for *Agrobacterium tumefaciens* A-47, *Erwinia amylovora* S59/5, *Erwinia carotovora spp. carotovora* SCC3193, *Pantoea agglomerans, Pseaudomonas syringae pv. atrofacience, Fusarium graminearum, Fusarium culmorum, Xanthomonas arboricola* S3, *Alternaria solani* K-100054, and *Phytophthora sp.*, and Corynebacterium Selective Agar for *Clavibacter michiganensis* VKM Ac-1404. The research team used 24-hour bacterial cultures and 7-day to 14-day fungal cultures. The final inocula contained 10^5^ CFU/mL for bacteria, and 1.1–1.5 × 10^2^ CFU/mL for fungi. For the control, the researchers used test tubes that contained nutrient media only.

Ten microliters of the inoculum (or a portion of the fungal mycelium) taken from tubes where no visible growth was observed were added using an inoculation loop to Petri dishes to find minimum bactericidal and fungicidal concentrations (MBC and MFC, respectively).

The results were recorded every 24 h over 5 days at 37 °C for *Staphylococcus aureus* 209P, 30 °C for *Agrobacterium tumefaciens* A-47, *Erwinia amylovora* S59/5, *Erwinia carotovora spp. carotovora* SCC3193, *Pantoea agglomerans*, and *Pseaudomonas syringae pv. atrofacience*, 28 °C for *Clavibacter michiganensis* VKM Ac-1404, and 25 °C for *Xanthomonas arboricola* S3. Fungal incubation in a thermostatically controlled chamber at 26 °C lasted 14 days with the corresponding substance. Microbial growth was detected visually [22]. All the tests were performed thrice.

### 2.6. Antioxidant Activity

The antiradical properties of ethanol extracts were tested by a chemiluminescent (CL) assay [23] using a Lum-100 chemiluminometer (DISoft, Moscow, Russia).

A 1 mmol/l luminol solution (Alfa Aesar, Heysham, Lancashire, UK UK) was obtained by dissolving in 0.1 M NaOH; before the assay, it was diluted 4-fold with distilled water. The reaction mix included: 400 μL of 250 μM luminol, 500 μL of 0.5 M Tris buffer solution (Fisher Chemical, Pittsburgh, UK) (pH 8.6), and 100 μL of 40 mM AAPH 2,2′-Azobis(2-methylpropionamidine) dihydrochloride (Acros Organics, Belgium, USA) in distilled water. The reaction mixture was incubated at 30 °C; the fundamental CL level was measured over 10 min. Then, 10 μL of the solution of the tested compound were added to the reaction mixture and the CL was measured over 20 to 30 min. The ethanol extract was diluted in distilled water to 10, 1, 0.1, and 0.01 mg/mL. The analysis was performed in triplicate.

Trolox (6-hydroxy-2,5,7,8-tetramethylchroman-2-carboxylic acid, Sigma-Aldrich Co., St. Louis, Missouri, USA) and quercetin were used as the standard antioxidants.

To compute the CL of the tested samples, the researchers calculated the TAR (total antioxidant response) and TRAP (total reactive antioxidant potential) [24].

By measuring the CL curve areas, the researchers were able to evaluate the relative inhibitory activity of each sample. The inhibition coefficients were calculated as per Equation (1):

(1) $$\mathrm{Inhibition}\left(\%\right)=\frac{100\cdot {\mathrm{AUC}}_{1}}{{\mathrm{AUC}}_{0}}$$

where AUC_0_ and AUC_1_ are areas under the curve for the control and for the tested solution, respectively.

The results were processed using PowerGraph (DISoft, Moscow, Russia) and OriginLab.

### 2.7. Total Polyphenol Content and Flavonoids

The ethanol extracts of *Centaurea jacea* L., *Centaurea cyanus* L., and *Centaurea scabiosa* L. were tested for the total phenolic content using the Folin–Ciocâlteu method.

One hundred microliters of the corresponding extract dilutions were oxidized with 900 μL of the Folin–Ciocâlteu reagent. The reagents were mixed, left for 5 min, and then neutralized with 2 mL of 7.5% sodium carbonate. Then the mixture was left for 1 h in a dark at room temperature. After that, the team recorded the optical density of the samples at 765 nm using a LEKI SS1207 spectrophotometer. The total phenolic content was calculated from the calibration curve and expressed in milligrams of equivalent gallic acid per gram of the material.

The total flavonoid content was found by using Stanković’s method. One milliliter of the corresponding extracts, each concentrated at 1 mL/mL and 1 mL of 2% AlCl_3_ solution, was dissolved in methanol. Test samples were obtained thrice; the samples were left at room temperature for 1 h. Optical density was identified using a spectrophotometer at λ_max_ = 415 nm. A similar measurement was taken for the quercetin solution as the standard in use. The calibration curve was plotted from the collected data. The flavonoid concentration was expressed in terms of quercetin equivalent [25]. The analysis was performed in triplicate.

### 2.8. Flavonoid Counts by High-Performance Liquid Chromatography

The High-Performance Liquid Chromatography (HPLC) analyses were performed using: the COSMOSIL C18-MS-II column (250 mm × 4.5 mm, 5 μm), a quaternary gradient pump, and an intelligent UV-Vis detector. The compounds were eluted with a gradient elution of the mobile phases A and B. Solvent A consisted of deionized water and solvent B consisted of acetonitrile (HPLC grade). The gradient elution program was set as follows: 5% B–40% B (50 min), 40% B–5% B (10 min), and 5% B isocratic (5 min). The injection volume for all samples was 20 μL. Flavonoids were monitored at 280 nm and 285 nm at a flow rate of 0.8 mL/min. All determinations were performed in triplicate.

Flavonoids were identified by matching the retention time and their spectral characteristics against those of standards, and the contents of flavonoids were determined using calibration curves. Each standard solution (0.1–2.0 μg/mL quercetin, 0.1–0.5 mg/mL rutine, 0.05–0.5 μg/mL hesperidin) was dissolved in acetonitrile and subjected to HPLC analysis. The calibration curves were constructed by plotting the average peak areas vs. the concentration of each analyte.

### 2.9. Phytotoxicity

Phytotoxicity was measured in laboratory experiments by testing seed germination rates, as well as through biometric readings including the length of sprouts and roots and the fresh biomass of sprouts and roots. The test culture was of garden cress (*Lepidium sativum* L., Vitaminchik variety). Seeds were not treated with protectants. Germination procedures followed [26]. A total of 100 seeds were tested in each case to determine germination rates. For control, the research team used sterile distilled water, while the experimental seeds were treated by soaking in the extracts of *Centaurea cyanus* L., *Centaurea jacea* L., and *Centaurea scabiosa* L. (1 mg/mL solutions) for 2 h. For comparisons, the research team used 96% ethanol, chemically pure methanol, chemically pure MTBE, and chemically pure petroleum ether. The tests were carried out in 4 repetitions.

### 2.10. Statistical Analysis

The results were summarized as the mean ± standard deviation (SD). The statistics software STATISTICA version 10 (StatSoft. Inc., Tulsa, OK, USA) was used to analyze differences among the 2 samples by applying a 2-tailed paired Student’s *t*-test at a 5% significance level. Significant differences between the samples were considered when the *p*-value was lower than 0.05. Tukey’s least significant difference was used for seed germination data to find significance between treatments at *p* < 0.05.

## 3. Results and Discussion

### 3.1. Phytochemical Composition of Flower Extracts for Some Centaurea L. Species

By using the gas chromatography/mass spectrometry (GC-MS) method, the authors were able to obtain new data on the phytochemical composition of freshly collected biomass from flowers of the genus *Centaurea* (*Centaurea cyanus* L.) – cornflower (*Centaurea cyanus* L.), brown knapweed (*Centaurea jacea* L.), and greater knapweed (*Centaurea scabiosa* L.), which are typical representatives of the flora from the middle belt of the Russian Federation. Studies of plants from the genus *Centaurea*, typical representatives of Central Russia’s flora, are rare [11,27]. In the vast majority of cases, species native to Turkey (e.g., *Centaurea antiochia* var., *Centaurea hypoleuca*, *Centaurea amaena* Boiss. & Balansa, *Centaurea aksoyi* Hamzaoglu & Budak, *Centaurea babylonica* L.) [13,14,15] and Africa (*Centaurea pumilio* L. [28] and 26 other species [4]) are studied.

To compare different *Centaurea* plants in terms of phytochemistry, the research team sampled ethanol extracts produced from freshly harvested *Centaurea cyanus* L., *Centaurea jacea* L., and *Centaurea scabiosa* L. flowers (see Table 1).

The phytochemical compositions of these extracts varied both qualitatively and quantitatively. *Centaurea jacea* L. had as few as 13 compounds, while *Centaurea scabiosa* L. had as many as 27. Palmitic acid and phytol were found in all the samples.

Catechol and α-amyrin (>10%) were the major components of the ethanol extract produced from *Centaurea cyanus* L. flowers.

The *Centaurea jacea* L. flower extract was dominated by 3′,5,6-trihydroxy-3,4′,7-trimethoxy flavone (>60%).

Ethanol extract produced from freshly harvested *Centaurea scabiosa* L. flowers (Table 2) contained 27 components distributed as follows: fatty carboxylic acids (29.10%), heterocyclic compounds (15.35%), esters (15.22%), an aromatic hydrazide (6.33%), a sesquiterpenoid (4.61%), a phenylpropanoid (4.45%), an aromatic ether (3.44%), a disaccharide (3.38%), an aromatic alcohol (2.93%), a terpenoid (2.93%), a diterpenol (1.64%), a sterol (1.64%), an aromatic bicyclic compound (1.53%), a flavonoid (1.47%), and a triterpene (0.45%).

Biologically active substances such as pyranone, coumaran (2,3-dihydrobenzofuran), and 5-hydroxymethylfurfural were first identified in the composition of *Centaurea scabiosa* L., as well as compounds previously described in the literature—phytol, chamazulene, stigmasterol, γ-sitosterol, and α-amyrin. They accounted for about 20% of the total number of identified components.

Based on the GC-MS literature data, an analysis of the phytochemical composition of knapweeds growing wild in the Russian Federation was carried out. The complete set of studies included ultraviolet, infrared spectroscopy, high-performance liquid chromatography (HPLC), chromatography–mass spectrometry, and ^1^H nuclear magnetic resonance spectroscopy for only one species – *Centaurea scabiosa* L. For cornflower and brown knapweed, studies of the phytochemical composition were performed mainly using the HPLC method on plants growing in Germany [9], Turkey [29,30], and Portugal [31].

Since the biomass of plants of the genus *Centaurea* contains a wide range of compounds of different chemical nature, it is advisable to use various polar and non-polar solvents to extract the maximum possible number of components. According to the literature, water, methanol, ethanol, butanol, ethyl acetate, hexane, chloroform, dichloromethane, or a mixture are most commonly used solvents [4,9,11,12,30].

Studies were conducted on the efficiency of extraction for freshly picked flowers of *Centaurea scabiosa* L. when used as an extractant of methanol. Petroleum ether and MTBE along with ethanol were used.

Chromatography of the methanol extract produced from freshly harvested flowers (Table 2) showed far fewer compounds, with only 13 components distributed as follows: aromatic ether (46.6%), heterocyclic compounds (33.46%), a disaccharide (6.37%), a lactone (5.32%), sesquiterpenoids (3.85%), fatty carboxylic acids (1.9%), trienol (1.09%), an ester (0.89%), and diterpenol (0.53%).

The methanol extract contained coumaran (2,3-dihydrobenzofuran), 5-hydroxymethylfurfural, 3-hydroxy-4-methoxybenzoic acid, methyl ester and 3-methyl-coumarin. These components accounted for 80% of the total of identified substances. Coumaran and 5-hydroxymethylfurfural were present in a greater quantity as compared to the ethanol extract.

Chromatography of the petroleum ether extract produced from freshly harvested flowers identified 39 components, mainly alkanes: 28.21% (Table 2). Other major compounds included fatty carboxylic acid ethers (17.38%), diterpenes (16.26%), sterols (13.68%), diterpenols (8.5%), fatty carboxylic acids (7.26%), diketones (2.12%), triterpenes (1.94%), a chlorinated aromatic compound (1.2%), and a high-molecular-weight alcohol (1.15%). Minor components of the extract included an aromatic acid ether (0.47%), an alkatriene (0.47%), an alkene (0.47%), a ketolactone (0.39%), an aromatic aldehyde (0.35%), and a ketosesquiterpene (0.17%).

As for individual components, γ-sitosterol, tetratriacontane, benzenepropanoic acid, 3,5-bis(1,1-dimethylethyl)-4-hydroxy-, and octadecyl ester were the most abundant, accounting for >7% of the total of components.

Chromatography showed that it was the MTBE extract of freshly harvested flowers that had the most diverse composition (see Table 2). Forty-three compounds were identified, distributed as follows: monoterpenol ester (38.42%), sesquiterpenes (20.81%), alkanes (19.67%), dieterpenes (4.83%), ethers and amides of fatty carboxylic acids (3.86%), triterpenes (3.83%), sterols (3.63%), vitamin A acetate (2.15%), ketones (1.23%), a phytohormone (1.17%), an alkene (0.62%), and an aromatic ether (0.18%).

L-bornyl acetate (38.42%) and caryophyllene (8.08%) were the most abundant individual compounds.

The extracts also contained compounds in concentrations of less than 1% of the total amount of components: (1) the ethanol extract —α-amyrin (0.45%); (2) the methanol extract – linoelaidic acid (0.91%); (3) the petroleum ether extract – heptacosyl acetate (0.98%), nonacos-1-ene (0.47%), tetracosanoic acid, methyl ester (0.63%), hentriacontane (0.49%), 7,9-di-tert-butyl-1-oxaspiro(4,5)deca-6,9-diene-2,8-dione (0.39%), benzoic acid, undecyl ester (0.47%), hexahydrofarnesyl acetone (0.17%), neophytadiene (0.67%), 1,E-11,Z-13-octadecatriene (0.47%), dodecanoic acid (0.42%); and (4) the MTBE extract – hexacosane (0.67%), chondrillast-7-enol (0.59%), β-amyrin acetate (0.38%), hexacosanoic acid, methyl ester (0.20%), nonacos-1-ene (0.62%), squalene (0.37%), linolelaidic acid, methyl ester (0.88%), palmitic acid, 2-hydroxyethyl ester (0.11%), isopimaral (0.22%), hexadecanamide (0.69%), palmitoleamide (0.14%), abieta-7,13-diene (0.24%), eicosanal (0.13%), ethyl palmitate (0.21%), n-hexadecanoic acid (0.99%), 4-biphenylcarboxaldehyde (0.35%), palmitic acid, methyl ester (0.17%), salvial-4(14)-en-1-one (0.10%), benzoic acid, pentyl ester (0.18%), heptadecane (0.27%), 2-n-butyl-2-cyclopentenone (0.73%), nonadecane (0.30%).

Ultra-high-performance liquid chromatography electrospray ionization/high-resolution mass spectrometry analysis of the MTBE extract of *Centaurea scabiosa* L. flowers was performed since it contained the largest number of individual compounds to assess the qualitative composition of the non-volatile components.

As a result of the analysis, 14 compounds were found in significant quantities, as shown in the following Table 3. No previously undetected components were identified among them [32]. The most common compounds for the Centaurea family plants were sesquiterpene lactones (cynaropicrin, grossgemin, repnin). Sesquiterpene lactones are typical components of plants in the Asteraceae family [3]. Flavones (apigenin, luteolin, lutein, flavonols), quercetin, and cinnamic acids were also identified. The presence of these compounds in the composition of *Centaurea scabiosa* L. and *Centaurea cyanus* L. are indicated by [1,11,31].

### 3.2. Antimicrobial Activity

Step 1 was to screen the antimicrobial activity of methanol extracts produced from freshly harvested flower biomass of the three tested *Centaurea* species; the team tested such activity against 1 human pathogenic bacterial strain, 6 phytopathogenic strains (2 Gram+ and 6 Gram-), and 4 phytopathogenic fungal strains.

The minimal inhibitory concentrations (MICs) of methanol extracts produced from *Centaurea jacea* L. and *Centaurea cyanus* L. were within 2500 to 5000 μg/mL. For *Centaurea scabiosa* L., these concentrations were lower at within 1250–2500 μg/mL, except against *Fusarium culmorum* (5000 μg/mL).

Minimum bactericidal concentrations for most of the tested microorganisms were 10,000 μg/mL or more, with the exception of *Clavibacter michiganensis* and *Alternaria solani*, which the methanol extract of *Centaurea scabiosa* L. was effective against at 2500 μg/mL.

The obtained values of the minimum inhibitory, minimum bactericidal, and fungicidal concentrations of flower extracts of the studied knapweeds were 10–4000 times higher than similar values for the reference compounds for bacteria (norfloxacin, chloramphenicol), or fungi (difenoconazole), indicating their low antimicrobial activity.

According to the literature, knapweed extracts for further use in medical, food, and other purposes are obtained in most cases from dried aboveground biomass (leaves, flowers) [4,11,31]. Stems are not used due to the presence of large numbers of ballast substances (cellulose, lignin). Extracts from fresh raw materials and the rapid freezing of raw materials have been much less studied. A comparative analysis of the extraction of different types of raw materials and extragents will determine the most optimal option that provides the highest yield and biological activity of the chemical components of plant extracts.

The next step was to study in detail the antimicrobial activity of extracts produced from freshly harvested and flash-frozen *Centaurea scabiosa* L. flowers. These extracts were prepared using water, ethanol, methanol, petroleum ether, and MTBE.

As test strains of microorganisms, the phytopathogenic bacterium *Clavibacter michiganensis* ICM Ac-1404 (a representative of Gram+ microorganisms) and the phytopathogenic fungus *Alternaria solani* St108, widely distributed pathogens that parasitize plants, were used. The results are presented in Table 4.

Flower extracts obtained with different extraction agents had different antimicrobial activity against phytopathogens. All the tested solvents can be ranked by the antimicrobial activity of the resulting *Centaurea scabiosa* L. in ascending order: water > ethanol 70% > methanol > petroleum ether > MTBE.

Extracts produced with MTBE were most effective against the tested phytopathogens, with MIC values between 60 and 120 μg/mL, MFCs of 120 μg/mL, and MBCs of 250 μg/mL. According to Van Vuuren and Holl (2017), and according to the most recent published criteria, when considering antimicrobial data, meaningful antimicrobial activity for plant extracts is considered at a minimum inhibitory concentration < 160 µg/mL [33].

Active antimicrobial components were best extracted by applying ethanol to flash-frozen plant biomass. Samples obtained by extracting freshly harvested flower biomass at room temperature were less antimicrobially active. Extraction by means of water, methanol, and MTBE in most cases showed no significant differences in the antimicrobial activity of the *Centaurea scabiosa* L. extracts. Petroleum ether produced the most active extracts when applied to freshly harvested flowers at room temperature.

The data in the literature suggest that antimicrobial activity in *Centaurea* species varies greatly from low (MIC > 20 mg/mL [9,14,15,28]) to high (MIC < 20 μg/mL) [13,34,35].

The increased antimicrobial activity of knapweed extracts is due to the presence in its composition of furan derivative—2,3-dihydrobenzofuran. Thus, the work of Sharafutdinov shows the bactericidal activity of chemically synthesized derivatives of 2 (5H)-furanone against gram-positive bacteria (*Staphylococcus aureus* [36] and *Bacillus subtilis* [37]). It was found that in the presence of 2(5H)-furanone derivatives, the formation of biofilms by gram-positive bacteria was suppressed, and cell death occurred at high concentrations of the compounds. The mechanism of the antimicrobial action of the 2(5H)-furanone derivative on *Staphylococcus aureus* consisted of a rapid penetration into cells, with the subsequent induction of oxidative stress and a direct interaction with a number of intracellular proteins, which led to a break in their structural and physicochemical properties and then cell death. In the case of gram-negative bacteria, no such effects were detected. Krasnov et al. (2012) found the water extract of *Centaurea scabiosa* L. to be markedly active against gram-positive bacteria *Staphylococcus aureus* and *Mycobacterium smegmatis.* They attributed that activity to sesquiterpene lactones [11].

### 3.3. Antioxidant Activity of Centaurea Ethanol Extracts

An essential characteristic of the biological properties of the components of plant extracts is their antioxidant properties. Currently, bioactive substances of plant origin in many countries are considered as an alternative to chemical preparations due to the relatively low cost of their production and the diverse side effects of synthetic substances.

Chemiluminescence analysis is standard among the methods for determining the content of antioxidants in plant objects. The total reactive antioxidant potential (TRAP) method is based on measuring the latent period of chemiluminescence and can be used to determine such antioxidants as Trolox. They are characterized by a high value of the reaction rate constant with radicals and, for this reason, can be called strong antioxidants. The TRAP value represents the latent period during which all the radicals react with the antioxidant. The total antioxidant reactivity (TAR) method measures the degree of chemiluminescence quenching at the plateau or the maximum of the chemiluminescence curve. The TAR value is comparable to the degree of quenching of the chemiluminescence intensity. It is believed that the TRAP reflects the number of antioxidants in the system and TAR shows its activity, i.e., the rate of interaction of the antioxidant with radicals.

**Figure 1:**
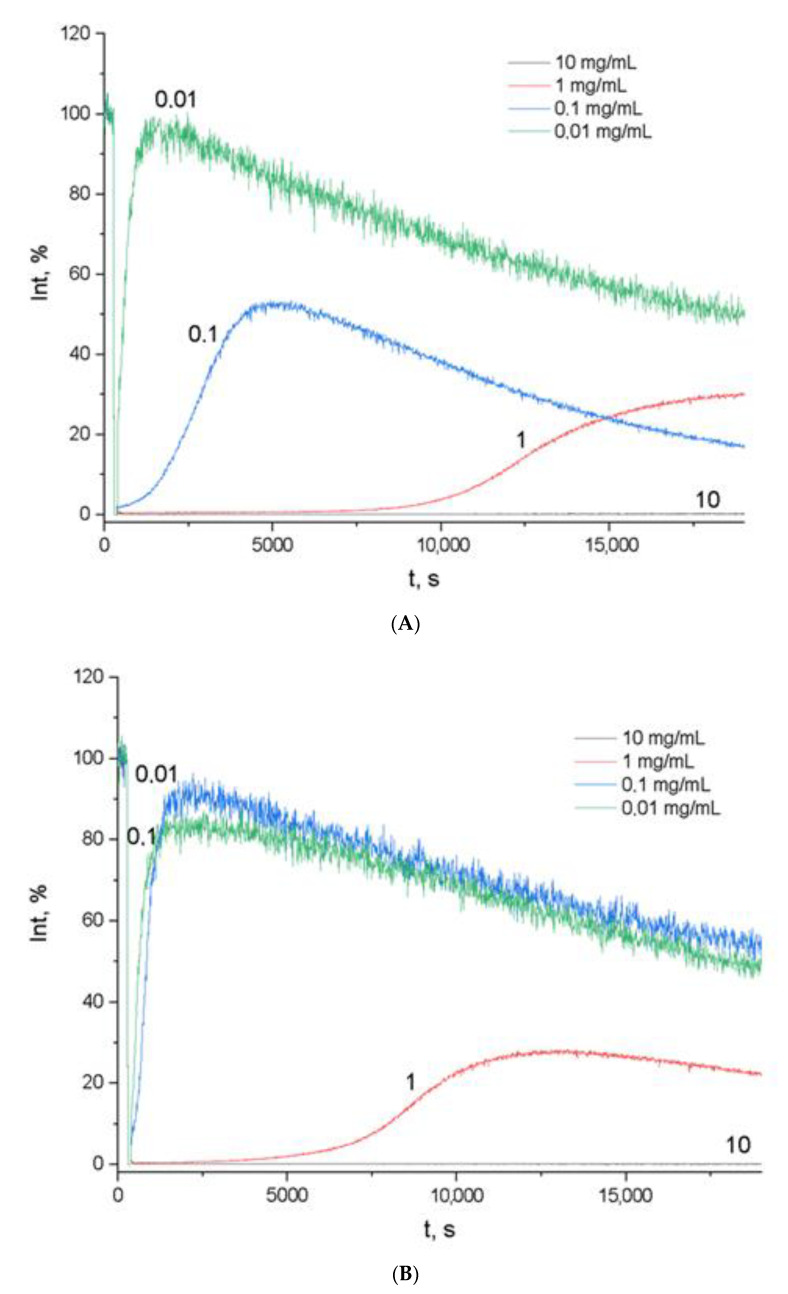

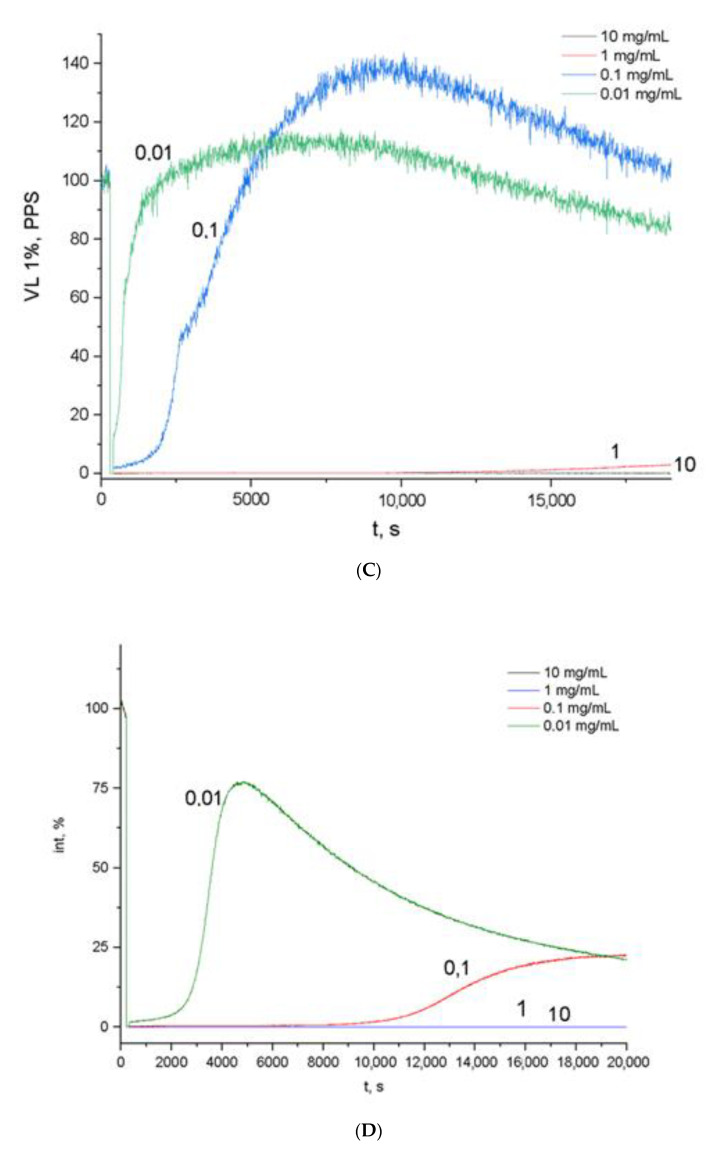

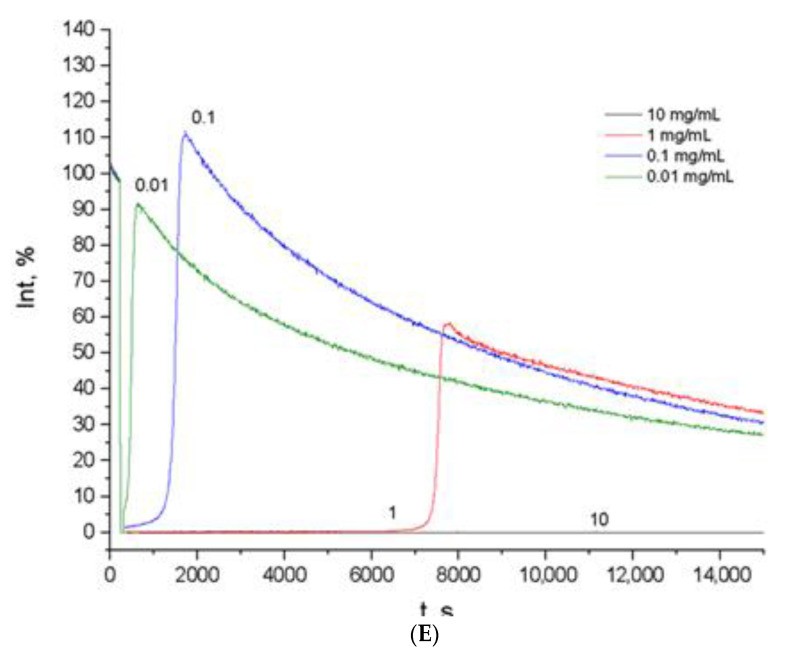
Chemiluminescence intensity vs. time of ethanol extracts from flowers of ***Centaurea cyanus* L.** (**A**), ***Centaurea scabiosa* L.** (**B**), ***Centaurea jacea* L.** (**C**), **quercetin** (**D**), and **Trolox** (**E**). The numbers beside the curves are the concentrations of *Centaurea* L. ethanol extract, dihydroquercetin, and Trolox (mg/mL). Time (s) is plotted on the abscissa axis and chemiluminescence intensity (a.u.) is plotted on the ordinate axis.

Chemiluminescent activity tests revealed weak antioxidant effects of ethanol extracts produced from the flowers of the tested *Centaurea* plants, with an effective concentration threshold of 0.01 mg/mL (Figure 1).

*Centaurea jacea* flowers (ethanol-extracted) were most capable of binding free radicals. *Centaurea cyanus* had a shorter latent period. *Centaurea scabiosa* had the weakest antioxidant response (Table 5).

Plant extracts of knapweed showed high levels of activity in the purification of radicals at a 10 mg/mL concentration, with TAR values of 99.75–99.93 and TRAP values of 19,000 s. The TRAP value for dihydroquercetin was 20,000 s, and for Trolox it was 19,000 s. When the concentration of plant substances decreased, the number of antioxidants in the system decreased, especially in *Centaurea scabiosa* L.

According to the TAR method, all the studied knapweed extracts at a concentration of 10 mg/mL consistently showed the ability to absorb free radicals in the system, in a manner similar to that of Trolox and dihydroquercetin (Table 6). This ability decreased with a decrease in the concentration of dry substances in the solution.

Kadyrova et al. (2014) also discovered relatively strong antioxidant effects exhibited by a 70% ethanol extract produced from *Centauria jacea* L. as compared to *Centaurea pseudomaculosa* Dobrocz; they used voltammetry for testing. At the same time, the authors noted that antioxidant activity and the concentration of flavonoids in extracts did correlate.

The authors hereof found that *Centauria jacea* L. flowers ranked first in this study in terms of the total flavonoid content and in terms of specific flavonoids (rutin, quercetin, hesperidin); the difference between this species and the other tested *Centauria* plants was a factor of 2.1 to 6.0 (Table 7).

An in-depth study was carried out on how the extractant and the sampling procedure affected the extraction of phenolic components from *Centauria scabiosa* L.; this study did not identify any significant difference in the total flavonoid content when using water, 70% ethanol, or chemically pure methanol, or when the extraction was from freshly harvested or liquid nitrogen flash-frozen flowers. When extracted with MTBE and petroleum ether, the total number of flavonoids was 4–8 times lower; this was probably due to their low solubility in non-polar solvents.

### 3.4. Phytotoxicity of Ethanol Extracts Produced from Centaurea L. Plants

**Figure 2:**
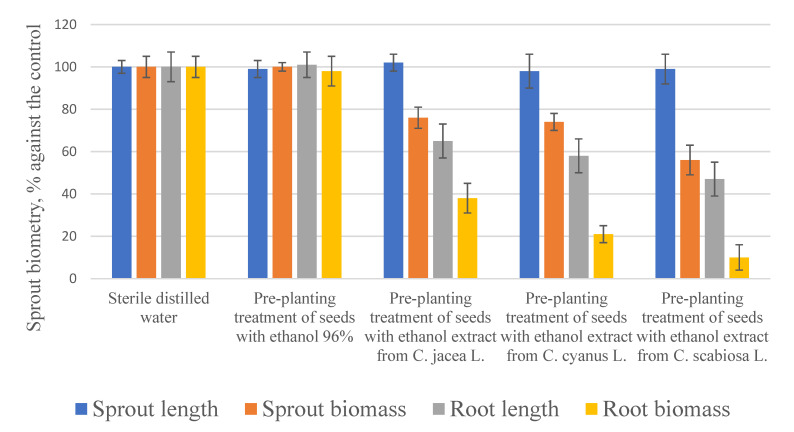
How pre-planting treatment of seeds with flower extracts (1 mg/mL) affected the biometry of garden cress sprouts compared to the controls.

There was no significant difference between the control (sterile distilled water) and the 96% ethanol variant in terms of seed germination rate and sprout biometry (Figure 2).

Plant extracts produced from freshly harvested/flash-frozen *Centaurea* flowers did not inhibit the germination of garden cress regardless of the extractant. No significant differences were found using Tukey’s test.

However, juvenile sprouting and rooting of extract-treated seeds were inhibited significantly compared to the controls.

The first step was to evaluate the toxicity of ethanol extracts produced from the three *Centaurea* plants (see Figure 2).

Pre-planting treatment did not affect the linear growth of sprouts; however, their biomass was 23% to 46% lower compared to the controls. Root growth was inhibited by 35% to 52% lengthwise, while the accumulation of fresh root biomass was even further depressed: by 64% to 89% compared to the controls. In terms of phytotoxicity, extracts can be ranked as follows in the ascending order: *Centauria jacea* L. > *Centauria cyanus* L. > *Centauria scabiosa* L.

The next step was to study how different extractants would affect the phytotoxicity of *Centauria scabiosa* L. extracts. Water extracts were the least toxic, as they depressed growth by 3–13% as a result of pre-planting treatment. The MTBE extract was most toxic, as it depressed the biometric indicators by 35–67%. No significant difference in phytotoxicity was found between the sample preparation methods. Root growth was most sensitive to the effects of the extract components.

## 4. Conclusions

New data on the phytochemical composition and biological activity of flower extracts of three plant species of the genus *Centaurea* were obtained in this study.

The phytochemical composition for ethanol extracts of flowers of the studied plant species of the genus *Centaurea*, identified using the GC-MS method, differed in the qualitative and quantitative composition of the components. The smallest number of compounds was identified in the extract of brown knapweed flowers, and the largest in greater knapweed.

Extracts obtained from the flowers of *Centaurea scabiosa* L. with the help of different solvents differed significantly in their composition. The largest number of components was identified by extraction with the use of the non-polar solvents petroleum ether and MTBE, with 39 and 43 compounds, respectively. For the first time, such biologically active substances as pyranone, coumaran (2,3-dihydrobenzofuran), and 5-hydroxymethylfurfural were identified in the ethanol and methanol extracts of *Centaurea scabiosa* L. by the GC-MS method.

At the screening stage, the low antimicrobial activity of methanol extracts of freshly harvested knapweed biomass against phytopathogenic microorganisms was established: the minimum inhibitory concentrations were 1250–10,000 µg/mL, while the minimum bactericidal and fungicidal concentrations were 2500 µg/mL or more. *Centaurea scabiosa* L. showed the greatest activity.

Extracts of *Centaurea scabiosa* L. flowers obtained using MTBE had the highest antimicrobial activity in relation to the studied phytopathogens: the minimum inhibitory concentrations were 60–120 µg/mL, the minimum fungicidal concentration was 120 µg/mL, and the minimum bactericidal concentration was 250 µg/mL.

The lowest antioxidant activity of the studied plants was found with the maximum values of *Centaurea jacea* L., which correlated with the content of individual flavonoids and the total content of flavonoids in the extracts. Knapweed extracts at a concentration of 0.1% showed no inhibitory effect on the germination of garden cress but did impact the growth of seedlings, with the most significant phytotoxic effect being that of greater knapweed.

## Figures and Tables

**Table 1:** Phytochemical composition of ethanol extracts produced from *Centaurea cyanus* L., *Centaurea jacea* L., and *Centaurea scabiosa* L. flowers.

Component	RR_t_	*Centaurea cyanus* L.	*Centaurea jacea* L.	*Centaurea scabiosa* L.
		% of the total		
Pyranone (heterocyclic chemical compounds)	3.69	nd	nd	6.29
Coumaran (furan)	4.65	nd	nd	1.23
Catechol (phenol)	4.98	17.79	5.65	nd
5-Hydroxymethylfurfural (furfural)	5.18	nd	nd	7.83
3-Ethyl-5-methylphenol (phenol)	8.98	nd	nd	2.93
2-Hydroxy-5-methylbenzaldehyde (aldehyde)	9.32–9.39	9.91	8.02	nd
4-Ethenyl-2-methoxyphenol (phenol)	6.51	nd	1.74	nd
Hydroquinone (phenol)	6.90	nd	2.21	nd
4-Methoxy-1-naphthol (phenol)	10.07	nd	nd	3.44
Caryophyllene oxide (sesquiterpenoid)	11.41	nd	0.81	nd
(1R,7S,E)-7-Isopropyl-4,10-dimethylenecyclodec-5-enol (sesquiterpenoid)	12.88	nd	nd	4.61
Sucrose (disaccharide)	13.86	nd	nd	3.38
4-((1E)-3-Hydroxy-1-propenyl)-2-methoxyphenol (phenol)	14.00–14.38	1.37	nd	4.45
Neophytadiene (diterpene)	15.87	1.71	nd	nd
14-Methyl pentadecanoic acid methyl ester (ester)	17.32	0.37	nd	nd
Hexadecanoic acid ethyl ester (ester)	18.00	nd	nd	1.12
Palmitic acid (fatty acid)	18.10	8.76	6.49	12.57
Phytol (diterpene alcohol)	20.17	4.15	1.47	1.64
Linoleic acid (fatty acid)	20.15	nd	nd	6.13
Linoleic acid ethyl ester (ester)	20.48	nd	nd	2.50
Linolenic acid ethyl ester (ester)	20.57	nd	nd	2.77
Linolenic acid (fatty acid)	20.77	8.02	2.71	5.01
[1,1’-Biphenyl]-2-ol acetate (aromatic compound)	21.24	nd	nd	8.58
4-(4-Hydroxyphenyl)benzohydrazide (hydrazide)	22.77	nd	nd	6.33
Chamazulene (aromatic compound)	23.05	nd	nd	1.53
5,8,11,14-Eicosatetraynoic acid (fatty acid)	24.67	nd	nd	5.35
Galangin flavanone (flavonoid)	24.90	nd	nd	1.47
2-Monopalmitin (alcohol)	25.56	4.49	nd	2.73
Palmitic acid β-monoglyceride (ester)	25.57	nd	1.15	nd
2-Monolinolein (glyceride)	27.28	nd	nd	2.60
Linolenic acid, 2-hydroxy-1-(hydroxymethyl)ethyl ester (Z,Z,Z)- (ester)	27.38	nd	nd	1.31
1-Monolinolein (glyceride)	27.72	4.34	0.77	nd
Eriostemin (flavonoid)	33.39	nd	2.73	nd
1,1,6-trimethyl-3-methylene-2-(3,6,9,13-tetramethyl-6-ethenye-10,14-dimethylene-pentadec-4-enyl)cyclohexane (alkene)	33.62	nd	nd	2.10
Stigmasterol (sterol)	33.70	4.87	nd	0.54
γ-Sitosterol (sterol)	34.29	8.64	nd	1.10
3′,5,6-trihydroxy-3,4′,7-trimethoxyflavone (flavonoid)	34.36	nd	60.42	nd
Vitexicarpin (flavonoid)	35.49	nd	5.83	nd
β-Amyrin (triterpene)	34.54	9.33	nd	nd
α-Amyrin (triterpene)	34.99	12.86	nd	0.45
Vitamin E acetate (vitamin)	35.65	3.39	nd	nd

nd: not detected.

**Table 2:** Phytochemical composition of different extracts produced from *Centaurea scabiosa* L. flowers.

Component	RR_t_	EtOH	MeOH	PE	MTBE
		% of the total			
Pyranone (heterocyclic chemical compounds)	3.69	6.29	nd	nd	nd
Coumaran (furan)	4.62–4.65	1.23	5.05	nd	nd
5-Hydroxymethylfurfural (furfural)	5.18–5.27	7.83	16.48	nd	nd
L-bornyl acetate (terpene ester)	5.55	nd	nd	nd	38.42
Caryophyllene (sesquiterpene)	7.98	nd	nd	nd	8.08
β-Yalangene (sesquiterpene)	8.05	nd	nd	5.32	nd
β-Copaene (sesquiterpene)	8.20	nd	nd	3.36	nd
Humulene (sesquiterpene)	8.61	nd	nd	nd	3.66
Isogermacrene D (sesquiterpene)	8.66	nd	nd	4.39	nd
3-Ethyl-5-methylphenol (phenol)	8.98	2.93	nd	nd	nd
D-Germacrene (sesquiterpene)	9.16	nd	nd	2.52	nd
β-Bisabolene (sesquiterpene)	9.68	nd	nd	nd	4.48
3-Hydroxy-4-methoxybenzoic acid, methyl ester (ester)	9.92	nd	46.60	nd	nd
4-Methoxy-1-naphthol (phenol)	10.07	3.44	nd	nd	nd
3-Methyl-coumarin (lactone)	10.36	nd	11.93	nd	nd
Germacrene D-4-ol (sesquiterpene alcohol)	10.98	nd	nd	3.30	nd
Caryophyllene oxide (sesquiterpenoid)	10.99	nd	nd	nd	2.89
Humulenol-II (sesquiterpene alcohol)	11.69	nd	1.84	nd	nd
2,6-Dimethyl-10-methylene-2,6,11-dodecatrienal (sesquiterpenoid)	11.91	nd	2.01	nd	nd
(1R,7S,E)-7-Isopropyl-4,10-dimethylenecyclodec-5-enol (sesquiterpenoid)	12.88–13.00	4.61	nd	5.20	nd
α-Bisabolol (sesquiterpene alcohol)	12.87	nd	nd	nd	1.60
Sucrose (disaccharide)	13.86–14.29	3.38	6.37	nd	nd
4-((1E)-3-Hydroxy-1-propenyl)-2-methoxyphenol (phenol)	14.00	4.45	nd	nd	nd
Palmitic acid (fatty acid)	17.66–18.10	12.57	nd	2.46	nd
Hexadecanoic acid ethyl ester (ester)	18.00	1.12	nd	nd	nd
3-Deoxy-d-mannoic lactone (lactone)	18.55	nd	5.32	nd	nd
2-Chlorofluorene (aromatic compound)	19.44	nd	nd	1.20	nd
Sclareol (diterpene alcohol)	19.75	nd	nd	nd	4.21
Phytol (diterpene alcohol)	19.75–20.17	1.64	0.53	nd	nd
Linoleic acid (fatty acid)	20.15	6.13	nd	nd	nd
(Z,Z,Z)-9,12,15-Octadecatrien-1-ol (alcohol)	20.21	nd	1.09	nd	nd
Linoleic acid ethyl ester (ester)	20.48	2.50	nd	nd	nd
Linolenic acid ethyl ester (ester)	20.57	2.77	nd	nd	0.40
Linolenic acid (fatty acid)	20.24–20.77	5.01	nd	1.14	nd
[1,1’-Biphenyl]-2-ol, acetate (aromatic compound)	21.24	8.58	nd	nd	nd
Tetracosane (alkane)	22.37–22.50	nd	nd	0.78	1.93
4-(4-Hydroxyphenyl)benzohydrazide (hydrazide)	22.77	6.33	nd	nd	nd
Chamazulene (aromatic compound)	23.05	1.53	nd	nd	nd
Linolenic acid, methyl ester (ester)	23.25	nd	nd	nd	1.06
Eicosanoic acid (fatty acid)	23.77	nd	nd	3.24	nd
5,8,11,14-Eicosatetraynoic acid (fatty acid)	24.67	5.35	nd	nd	nd
Galangin flavanone (flavonoid)	24.90	1.47	nd	nd	nd
Pentacosane (alkane)	24.99–25.13	nd	nd	1.46	1.43
2-Monopalmitin (alcohol)	25.13–25.56	2.73	nd	0.46	nd
Vitamin A acetate (vitamin)	26.32	nd	nd	nd	2.15
2-Monolinolein (glyceride)	27.26–50.40	2.60	0.89	5.89	nd
Linolenic acid, 2-hydroxy-1-(hydroxymethyl)ethyl ester (Z,Z,Z)-(ester)	27.38	1.31	nd	nd	nd
Octacosane (alkane)	27.42–28.65	nd	nd	1.05	4.17
Desogestrel (hormone)	28.06	nd	nd	nd	1.17
Tetratetracontane (alkane)	29.69	nd	nd	nd	6.36
Tetratriacontane (alkane)	29.89	nd	nd	19.71	nd
Heptacosane-6,8-dione (ketone)	30.79	nd	nd	1.00	nd
Octacosanol (alcohol)	31.65	nd	nd	1.15	nd
Hentriacontane (alkane)	31.83	nd	nd	nd	4.41
Tetratriacontane (alkane)	32.00	nd	nd	4.72	nd
Nonacosane-6,8-dione (ketone)	32.78–32.98	nd	nd	1.12	0.50
Campesterol (sterol)	33.14	nd	nd	1.18	nd
Stigmasterol (sterol)	33.28–33.53	0.54	nd	3.24	nd
1,1,6-trimethyl-3-methylene-2-(3,6,9,13-tetramethyl-6-ethenye-10,14-dimethylene-pentadec-4-enyl)cyclohexane (alkene)	33.62	2.10	nd	nd	nd
Canophyllal (triterpenoid)	33.64	nd	nd	nd	1.93
β-Sitosterol (sterol)	33.87	nd	nd	nd	3.04
γ-Sitosterol (sterol)	33.87–34.29	1.10	nd	9.26	nd
β-Amyrin (triterpene)	34.12–34.46	nd	nd	1.43	0.91
Benzenepropanoic acid, 3,5-bis(1,1-dimethylethyl)-4-hydroxy-, octadecyl ester (ester)	37.47	nd	nd	7.67	nd
Phytyl decanoate (ester)	43.86	nd	nd	1.75	nd

nd: not detected.

**Table 3:** Phytochemical composition of MTBE extracts produced from *Centaurea scabiosa L.* flowers.

Peak No.	[M-H]-m/z Molecular Formula	Proposed Compound
1	153.0182 C7H5O4	Protocatechuic acid (phenolic acid)
2	353.0885 C16H17O9	3-Caffeoylquinic acid (flavonoid ester)
3	353.0893 C16H17O9	5-Caffeoylquinic acid (flavonoid ester)
4	179.0342 C9H7O4	Caffeic acid (phenolic acid)
5	195.1981 C12H20O2	L-bornyl acetate (terpene ester)
6	463.0885 C21H19O12	Quercetin-3-O-glucoside (flavonoid)
7	593.1512 C27H29O15	Luteolin-7-O-rutinoside (flavonoid)
8	447.0926 C21H19O11	Luteolin-7-O-glucoside (flavonoid)
9	301.0353 C15H9O7	Quercetin (flavonoid)
10	269.0453 C15H9O6	Apigenin (flavonoid)
11	C19H21O7 361.0721	Repin (flavonoid)
12	285.0401 C15H9O6	Luteolin (flavonoid)
13	333.0126 C18H21O6	Cynaropikrin (sesquiterpene lactone)
14	263.0328 C15H19O4	Grossgemin (sesquiterpene lactone)

**Table 4:** Antimicrobial activity of extracts produced from freshly harvested and flash-frozen *Centaurea scabiosa* L. flowers extracted by various solvents (μg/mL).

Solvents, Raw for Extraction	*Clavibacter michiganensis* VKM Ac-1404		*Alternaria solani K*-100054		*Statistics ^a^*
	MIC	MBC	MIC	MBC	
Ethanol, freshly harvested flowers	1250 ± 100	5000 ± 300	2500 ± 200	>10,000	0.001
Ethanol, flash-frozen flowers	625 ± 50	2500 ± 200	1250 ± 100	5000 ± 500	<0.001
Water, freshly harvested flowers	5000 ± 300	>10,000	5000 ± 300	>10,000	0.001
Water, flash-frozen flowers	5000 ± 300	>10000	5000 ± 300	>10000	0.001
Petroleum ether, freshly harvested flowers	120 ± 10	500 ± 30	120 ± 10	250 ± 20	<0.001
Petroleum ether, flash-frozen flowers	120 ± 10	1000±100	250 ± 20	500 ± 100	<0.001
MTBE, freshly harvested flowers	120 ± 10	250 ± 20	60 ± 6	120 ± 10	<0.001
MTBE, flash-frozen flowers	60±6	250 ± 30	60 ± 6	120 ± 10	<0.001

^a^ Statistical differences (*p* < 0.05) were assessed by applying a 2-tailed paired Student’s *t*-test.

**Table 5:** TRAP of ethanol extract from *Centaurea* L. flowers.

Species	10 mg/mL	1 mg/mL	0.1 mg/mL	0.01 mg/mL
*Centaurea cyanus* L.	19,000	6576	220	0
*Centaurea jacea* L.	19,000	9212	179	0
*Centaurea scabiosa* L.	19,000	3572	90	0
Trolox	19,000	14,000	2697	443
Dihydroquercetin	20,000	16,152	14,647	922

**Table 6:** TAR of ethanol extract from *Centaurea* L. flowers.

Species	10 mg/mL	1 mg/mL	0.1 mg/mL	0.01 mg/mL
*Centaurea cyanus* L.	99.75	70.06	47.75	2.16
*Centaurea jacea* L.	99.79	96.9	−38.4	−13.31
*Centaurea scabiosa* L.	99.93	72.52	7.97	16.06
Trolox	99.96	99.77	4.5	1.68
Dihydroquercetin	99.99	99.19	96.83	30.37

**Table 7:** Quantification of flavonoids and their total content in the flowers of 3 *Centaurea* species extracted by 70% ethanol.

Species	Rutin, mg/L	Quercetin, mg/L	Hesperidin, mg/L	Total Phenolic Content ^a^, mg GAE/l	Total Flavonoid Content ^b^, mg Kv/l	Statistics ^c^
*Centaurea cyanus* L.	13.2 ± 0.03	5.6 ± 0.01	13.2 ± 0.02	97.9 ± 1.15	118.52 ± 3.11	<0.001
*Centaurea jacea* L.	52.3 ± 1.72	11.9 ± 0.07	28.9 ± 0.23	163.7 ± 2.25	193.63 ± 2.67	0.001
*Centaurea scabiosa* L.	11.4 ± 0.19	3.4 ± 0.01	9.7 ± 0.03	91.3 ± 1.07	97.21 ± 1.00	<0.001

^a^ total phenolic content given as gallic acid equivalents (GAE). ^b^ total flavonoid content in terms of quercetin (Kv). ^c^ Statistical differences (*p* < 0.05) were assessed by applying a 2-tailed paired Student’s *t*-test.

## References

[1] Khammar A., Djeddi S. (2012). Pharmacological and biological properties of some *Centaurea* species. Eur. J. Res..

[2] Joujeh R., Zaid S., Mona S. (2019). Pollen morphology of some selected species of the genus *Centaurea* L. (Asteraceae) from Syria. S. Afr. J. Bot..

[3] Rustaiyan A., Faridchehr A. (2020). Constituents and biological activities of selected genera of the Iranian Asteraceae family. J. Herb. Med..

[4] Ayad R., Akkal S., Atta-ur-Rahman (2019). Phytochemistry and biological activities of algerian *Centaurea* and related genera. Studies in Natural Products Chemistry.

[5] Bouafia M., Benarfa A., Gourine N., Yousfi M. (2020). Seasonal variation of fatty acid composition, tocopherol content and antioxidant activity of lipid extracts from *Centaurea* sp. Food Biosci..

[6] Cherepanov S.K., Tsvelev N.N., Klokov M.V., Sosnovsky D.I., Komarova V.L., Bobrov E.G., Cherepanov S.K. (1963). Genus 1624. Cornflower—Centaurea. Flora of the USSR: In 30 Volumes.

[7] Aktumsek A., Zengin G., Guler G., Cakmak Y., Duran A. (2011). Screening for in vitro antioxidant properties and fatty acid profiles of five *Centaurea* L. species from Turkey flora. Food Chem. Toxicol..

[8] Koc S., Isgor B.S., Isgor Y.G., Moghaddam Sh N., Yildirim O. (2015). The potential medicinal value of plants from Asteraceae family with antioxidant defense enzymes as biological targets. Pharm. Biol..

[9] Lockowandt L., Pinela J., Roriz C.L., Pereira C., Abreu R.M.V., Calhelha R.C., Alves M.J., Barros L., Bredol M., Ferreira I.C.F.R. (2019). Chemical features and bioactivities of cornflower (*Centaurea cyanus* L.) capitula: The blue flowers and the unexplored non-edible part. Ind. Crop Prod..

[10] Gürağaç Dereli F.T., Ilhan M., Küpeli Akkol E. (2020). Identification of the main active antidepressant constituents in a traditional Turkish medicinal plant, *Centaurea kurdica* Reichardt. J. Ethnopharmacol..

[11] Krasnov E.A., Kaminskij I.P., Kadyrova T.V., Pekhen’ko V.G., Adekenov S.M. (2012). Antimicrobial activity of extracts from the aerial part *Centaurea scabiosa* (Asteraceae). Rastit. Resur..

[12] Grienke U., Brkanac S.R., Vujčić V., Urban E., Ivanković S., Stojković R., Rollinger J.M., Kralj J., Brozovic A., Radić Stojković M. (2018). Biological activity of flavonoids and rare sesquiterpene lactones isolated from *Centaurea ragusina* L.. Front Pharmacol..

[13] Albayrak S., Atasagun B., Aksoy A. (2017). Comparison of phenolic components and biological activities of two *Centaurea* sp. obtained by three extraction techniques. Asian Pac. J. Trop. Med..

[14] Özcan K., Acet T., Çorbacı C. (2019). *Centaurea hypoleuca* DC: Phenolic content, antimicrobial, antioxidant and enzyme inhibitory activities. S. Afr. J. Bot..

[15] Güvensen N.C., Keskin D., Güneş H., Oktay M.K., Yıldırım H. (2019). Antimicrobial property and antiproliferative activity of *Centaurea babylonica* (L.) L. on human carcinomas and cervical cancer cell lines. Ann. Agric. Environ. Med..

[16] Naeim H., El-Hawiet A., Abdel Rahman R.A., Hussein A., Demellawy M.A., Embaby A.M. (2020). Antibacterial activity of *Centaurea pumilio* L. root and aerial part extracts against some multidrug resistant bacteria. BMC Complem. Altern. M.

[17] ISO/IEC GUIDE 98-1:2009 (2009). Uncertainty of Measurement—Part 1: Introduction to the Expression of Uncertainty in Measurement.

[18] Fitsev I., Shlyamina O., Makaeva A., Nasybullina G., Saifutdinov A. (2020). Detection of cypermethrin residues in toxicological control objects using gas chromatography-mass spectrometry with solid-phase extraction. Int. J. Mech. Prod. Eng. Res. Dev..

[19] Clinical and Laboratory Standards Institutes (CLSI) (2018). Methods for dilution antimicrobial susceptibility tests for bacteria that grow aerobically. CLSI Standard M07.

[20] Kanagarajan V., Ezhilarasi M.R., Gopalakrishnan M. (2011). In vitro microbiological evaluation of 1,1′-(5,5′-(1,4-phenylene)bis(3-aryl-1H-pyrazole-5,1-(4H,5H)-diyl))diethanones, novel bisacetylated pyrazoles. Org. Med. Chem. Lett..

[21] Clinical and Laboratory Standards Institutes (CLSI) (2017). Reference method for broth dilution antifungal susceptibility testing of yeasts. CLSI Standard M27.

[22] Semenov V.E., Voloshina A.D., Kulik N.V., Strobykina A.S., Giniyatullin R.K., Saifina L.F., Nikolaev A.E., Krylova E.S., Zobov V.V., Reznik V.S. (2015). Macrocyclic and acyclic 1,3-bis5-(trialkylammonio)pentyl-5(6)-substituted uracil dibromides: Synthesis, antimicrobial properties, and the structure-activity relationship. Russ. Chem. Bull..

[23] Vyshtakalyuk A.B., Semenov V.E., Sudakov I.A., Bushmeleva K.N., Gumarova L.F., Parfenov A.A., Nazarov N.G., Galyametdinova I.V., Zobov V. (2018). Xymedon conjugate with biogenic acids. Antioxidant properties of a conjugate of Xymedon with L-ascorbic acid. Russ. Chem. Bull..

[24] Desmarchelier C., Repetto M., Coussio J., Llesuy S., Ciccia G. (1997). Total reactive antioxidant potential (TRAP) and total antioxidant reactivity (TAR) of medicinal plants used in southwest Amazonia (Bolivia and Peru). Int. J. Pharmacogn..

[25] Stanković M.S. (2011). Total phenolic content, flavonoid concentration and antioxidant activity of *Marrubium peregrinum* L. extracts. Kragujev. J. Sci..

[26] GOST 12038-84 (2004). Seeds of crops. Germination Methods.

[27] Kadyrova T.V., Ermilova E.V., Larkina M.S. (2014). Antioxidant activity of extracts of *Centaurea jacea* L. and *Centaurea pseudomaculosa* Dobrocz. Chem. Plant Raw Mater..

[28] Zater H., Huet J., Fontaine V., Benayache S., Stévigny C., Duez P., Benayache F. (2016). Chemical constituents, cytotoxic, antifungal and antimicrobial properties of *Centaurea diluta* Ait. subsp. algeriensis (Coss. & Dur.) Maire. Asian Pac. J. Trop. Med..

[29] Zengin G., Zheleva-Dimitrova D., Gevrenova R., Nedialkov P., Mocan A., Ćirić A., Glamočlija J., Soković M., Aktumsek A., Mahomoodally M.F. (2018). Identification of phenolic components via LC-MS analysis and biological activities of two Centaurea species: *C. drabifolia* subsp. *drabifolia* and *C. lycopifolia*. J. Pharm. Biomed. Anal..

[30] Uysal A., Zengin G., Mahomoodally M.F., Picot-Allain C., Jekő J., Cziáky Z., Rodrigues M.J., Ak G., Polat R., Urusan Z. (2021). A comparative study on biological properties and chemical profiles of different solvent extracts from *Centaurea bingoelensis*, an endemic plant of Turkey. Process. Biochem..

[31] Fernandes L., Pereira J.A., Saraiva J.A., Ramalhosa E., Casal S. (2019). Phytochemical characterization of *Borago officinalis* L. and *Centaurea cyanus* L. during flower development. Food Res. Int..

[32] Kaminsky I.P., Kadyrova T.V., Kalinkina G.I., Larkina M.S., Ermilova E.V., Belousov M.V. (2020). Comparative pharmacognostic study of rough cornflower (*Centaurea scabiosa* L.) growing wild and cultivated in the conditions of Tomsk. Chem. Plant Raw Mater..

[33] Van Vuuren S., Holl D. (2017). Antimicrobial natural product research: A review from a South African perspective for the years 2009–2016. J. Ethnopharmacol..

[34] Sarker S.K., Shoeb M., Celik S., Yucel E., Middleton M., Nahar L. (2005). Antibacterial and antioxidant activities of three Turkish species of the genus Centaurea. Orient Pharm. Exp. Med..

[35] Al-Saleem M.S., Awaad A.S., Alothman M.R., Alqasoumi S.I. (2018). Phytochemical standardization and biological activities of certain desert plants growing in Saudi Arabia. Saudi Pharm. J..

[36] Sharafutdinov I.S., Pavlova A.S., Akhatova F.S., Khabibrakhmanova A.M., Rozhina E.V., Romanova Y.J., Fakhrullin R.F., Lodochnikova O.A., Kurbangalieva A.R., Bogachev M.I. (2019). Unraveling the molecular mechanism of selective antimicrobial activity of 2(5H)-furanone derivative against *Staphylococcus aureus*. Int. J. Mol. Sci..

[37] Trizna E.Y., Khakimullina E.N., Latypova L.Z., Kurbangalieva A.R., Sharafutdinov I.S., Evtyugin V.G., Babynin E.V., Bogachev M.I., Kayumov A.R. (2015). Thio derivatives of 2(5H)-Furanone as inhibitors against *Bacillus subtilis* biofilms. Acta Nat..

